# Localized retroperitoneal Castleman’s disease: a case report and review of the literature

**DOI:** 10.1186/1752-1947-8-93

**Published:** 2014-03-10

**Authors:** Rodrigo Aguilar-Rodriguez, Sorin-Lucian Milea, Ilhan Demirci, Susanne Herold, Michael Flasshove, Bernd Klosterhalfen, Horst Kinkel, Hermann Janßen

**Affiliations:** 1 Department of General, Visceral, Vascular and Thoracic Surgery, Hospital of Düren, Düren, Germany; 2 Department of Internal Medicine II, University of Gießen Lung Center, Gießen, Germany; 3 Department of Hematology and Internal Oncology, Hospital of Düren, Düren, Germany; 4 The Institute of Pathology, Hospital of Düren, Düren, Germany; 5 Department of Gastroenterology, Hepatology and Diabetology, Hospital of Düren, Düren, Germany

**Keywords:** Castleman’s disease, Hyaline vascular variant, Surgery, Unicentric

## Abstract

**Introduction:**

Castleman’s disease, also known as angiofollicular lymph node hyperplasia, is a rare disease with two known expansion types, unicentric and multicentric, which play a major role in determining therapy. We focus here on the unicentric type, which can be treated and cured by surgery. To date, approximately 1000 cases of Castleman’s disease have been reported in the literature.

**Case presentation:**

A 50-year-old Caucasian woman presented to our Department of Hematology and Internal Oncology with increasing fatigue as her sole symptom. Diagnostic investigations including laboratory studies, ultrasound, computed tomography and magnetic resonance imaging were performed. These revealed an interaortocaval, retroperitoneal tumor mass in her upper abdomen as the only manifestation of the disease. No enlarged lymph nodes were detected. We conducted a laparotomy with radical extirpation of the tumor mass (10×9×5.7cm). Complete tumor resection with clear margins was achieved. A pathological analysis of the resected sample showed atypical lymphoid tissue of small to medium cells with some clearly visible nucleoli, enlarged sinusoidal vessels, pleomorphic calcifications and focally preserved germinal-center-like structures. Histological and immunohistochemical analysis confirmed the diagnosis of Castleman’s disease: staining for CD3, CD5, CD10, CD20, CD23, CD79 and Ki-67 was strongly positive in the germinal-center-like structures. Histological findings clearly showed the disease to be the hyaline vascular subtype. Staining for cyclin D1 and CD30 was negative. Expression of CD15 was positive in the enlarged sinusoidal vessels. A supplementary clonality analysis was without pathological findings. Tests for human immunodeficiency virus and human herpes virus 8 were negative and results from a bone marrow biopsy were normal. Our patient recovered well from surgery and was discharged from our hospital. To date, no recurrence of the disease has been detected.

**Conclusion:**

Castleman’s disease is a rare disorder that remains a diagnostic challenge. Radical surgical resection is considered to be the gold standard for treating the unicentric variant of this disease.

## Introduction

Castleman’s disease, also known as angiofollicular lymph node hyperplasia, is a rare disorder first described by Benjamin Castleman in the mid-1950s. There are two known expansion types of this disease, unicentric and multicentric, which play a major role in determining therapy. We focus here on the unicentric type, which can be treated and cured by surgery. Because of the rarity of this disease, epidemiological data like prevalence or incidence are not available. The average age of patients with unicentric Castleman’s disease is around 30 to 40 years old. Patients with the multicentric form are usually around 50 to 60 years old. No dispositional age, race or sex seems to exist but the disease is widely associated with human herpes virus 8 (HHV-8) and human immunodeficiency virus (HIV) infection [1-4]. To date, approximately 1000 cases of Castleman’s disease have been reported in the literature [5].

## Case presentation

**Figure 1:**
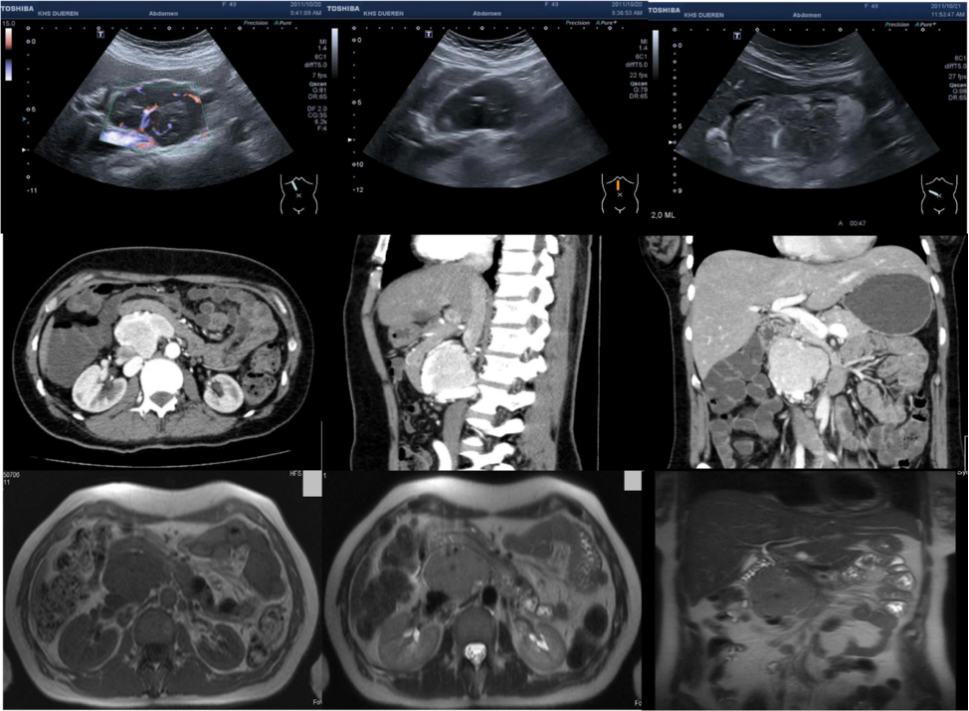
Pre-operative ultrasound, computed tomography and magnetic resonance imaging scans showing an interaortocaval tumor mass of approximately 6.3×4.7×6.8cm (measured on computed tomography scan).

**Figure 2:**
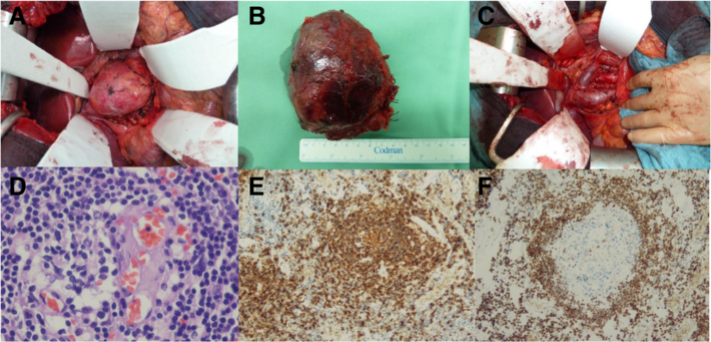
**Intra-operative pictures of the tumor *in situ *and after resection.** Hematoxylin and eosin staining and exemplary immunohistochemical staining for CD20 and for CD3. **(A, C)** Intraoperative pictures of the tumor in situ. **(B)** Resected tumor mass. **(D)** Hematoxylin and eosin staining showing hyaline vascular subtype. **(E)** Immunohistochemical staining for CD20. **(F)** Immunohistochemical staining for CD3.

In October 2011, a 50-year-old Caucasian woman presented to our Department of Hematology and Internal Oncology with increasing fatigue as her sole symptom. Diagnostic investigations including laboratory studies, ultrasound, computed tomography (CT) and magnetic resonance imaging (MRI) were performed. These revealed an interaortocaval, retroperitoneal tumor mass in her upper abdomen as the only manifestation of the disease (Figure 1). No enlarged lymph nodes were detected. We conducted a laparotomy with radical extirpation of a tumor mass of approximately 10×9×5.7cm and unknown dignity (Figure 2). Complete tumor resection with clear margins was achieved.

A pathological analysis showed atypical lymphoid tissue of small to medium cells with some clearly visible nucleoli, enlarged sinusoidal vessels, pleomorphic calcifications and focally preserved germinal-center-like structures. Histological and immunohistochemical analysis confirmed the diagnosis of Castleman’s disease (see Figure 2): staining for CD3, CD5, CD10, CD20, CD23, CD79 and Ki-67 was strongly positive in the germinal-center-like structures. Histological findings clearly showed the disease to be the hyaline vascular subtype. Staining for cyclin D1 and CD 30 was negative. Expression of CD15 was positive in the above-mentioned enlarged sinusoidal vessels. A supplementary clonality analysis was without pathological findings. Tests for HIV and HHV-8 were negative and results of a bone marrow biopsy were normal. Our patient recovered well from the surgery and was discharged from our hospital in early November. Follow-up care was carried out by our Department of Hematology and Internal Oncology. At the time of writing, no recurrence of the disease had been detected.

## Discussion

The histological subtypes of Castleman’s disease are as follows: hyaline vascular variant (unicentric in 72% of all cases); plasma cell variant (unicentric in 18% and multicentric in 10% of all cases); mixed variant, and a plasmablastic variant of multicentric Castleman’s disease [3,4,6-10]. The disease may occur anywhere in the lymphatic system but is found in the thorax in 60% to 70% of cases. The mediastinum is the most common localization for the unicentric type; a retroperitoneal multicentric type is among the rarest. Only 10% to 17% of cases involve the abdomen, of which the majority of cases are retroperitoneal [3,11-14] (Table 1). The plasmablastic variant of the disease is commonly associated with HHV-8 and tends to have a poor prognosis. It occurs more frequently in patients who are HIV positive, although there seems to be no correlation with CD4 count [15]. The multicentric and, especially, HHV-8-positive types are risk factors for the development of Hodgkin lymphoma, non-Hodgkin lymphoma and Kaposi sarcoma. Non-Hodgkin lymphomas are mainly characterized as B-cell lymphoma subtypes (71%). Less frequently associated non-Hodgkin lymphomas are plasmocytoma and T-cell lymphoma [5,16,17]. In a 2009 study, Fazakas *et al*. supposed a possible etiological relationship between a coexistent HHV-6 infection and multicentric plasmocytic Castleman’s disease with POEMS syndrome (polyneuropathy, organomegaly, endocrinopathy, monoclonal gammopathy and skin changes) [18].

**Table 1:** Overview of the histological variants and sex and age distribution of localized retroperitoneal Castleman’s disease in the literature

**Histological variants**				**Distribution of sex**			**Distribution of age (in years)**			
**HV**	**PC**	**Mixed**	**NK**	**M**	**F**	**NK**	**Age**	**M**	**F**	**NK**
148	9	5	37	69	79	51	0 to 9	2	0	52
74.37%	4.52%	2.51%	18.59%	34.67%	39.7%	25.63%	10 to 19	9	6	
							20 to 29	7	21	
							30 to 39	16	18	
							40 to 49	14	13	
							50 to 59	7	7	
							60 to 69	12	3	
							70 to 79	1	0	
**In total: 199 cases**				**M:F ratio: 1:1.14**			**Mean**	**38.25**		

F, female; HV, hyaline vascular; M, male; NK, not known; PC, plasma cell.

The extent of clinical symptoms may vary widely. Unspecific symptoms like asymptomatic to symptomatic lymphadenopathy accompanied by fever, anemia, fatigue, abdominal or thoracic pain, and weight loss can be observed. Cutaneous symptoms like paraneoplastic pemphigus, erythema multiforme or lichen planus have been described. POEMS syndrome is also known to be associated with the disease, commonly with HHV-8-positive forms.

Laboratory studies with blood counts and measurement of C-reactive protein, interleukin 6 and liver function should be conducted. In addition, HIV and HHV-8 testing should be performed. In this context, it is interesting to note that false-positive D-dimer and negative fibrin(ogen) degradation product levels have been described in the literature [19]. Although pathological analysis still remains the gold standard for diagnosis, MRI, contrast-enhanced sonography, angiography and CT are valuable diagnostic tools [20]. MRI can be helpful in identifying the hypervascular appearance of the hyaline vascular type [21]. As Michail *et al*. stated, Castleman’s disease should be included in the differential diagnosis of any hypervascular and heterogeneous tumor mass in the retroperitoneum [22,23]. In this regard, one should note a recently published case report of unicentric hyaline vascular Castleman’s disease mimicking a paraspinal schwannoma [24].

Kim *et al*. described two distinctive types of radiological manifestation in abdominal Castleman’s disease: a localized type with single or multiple discrete masses, and a disseminated type with non-specific organomegaly and lymphadenopathy [25]. Differences in enhancement between the plasma cell and hyaline vascular variants on CT scans have been described previously. The hyaline vascular variant is known for its high enhancement on CT imaging, whereas the plasma cell variant shows only low enhancement. However, Lu and Wu pointed out that the degree of enhancement cannot be used as a reliable and specific diagnostic criterion to distinguish between the above-mentioned types [13]. In a recently published study, 'rim-like’ enhancement, a predominantly left-sided location in the retroperitoneum, and the presence of peritoneal thickening were described as relatively characteristic CT findings of the disease. Furthermore, non-specific features like necrosis, fibrosis and calcifications can be observed [14]. A fluorine-18-fluorodeoxyglucose positron emission tomography scan can be helpful in staging of the disease, but its role is still controversial.

Castleman’s disease remains difficult to diagnose preoperatively because the disease may resemble other malignant tumors. Therefore, exclusion of other diseases with lymphadenopathy, for example lupus erythematodes, thymoma, sarcoma, rheumatoid arthritis, HIV infection and lymphoma, is important for differential diagnosis [3]. According to Deschênes *et al*., fine-needle aspiration can be useful in diagnosing Castleman’s disease [26]. The unicentric type - if resected completely - is not associated with increased mortality and is known to have an excellent prognosis (Table 2). Therefore, radical surgical resection is considered to be the gold standard therapy in these cases. According to a recently published study, there are no differences in the surgical outcomes of deep and superficial Castleman’s disease as long as the resection is complete [27]. The prognosis and outcome of the multicentric type depend on many factors, like progression rate, infections and comorbidities, and tend to be poorer [2,4,6]. There are no clear data about how long follow-up care should be conducted. Patients treated with radiotherapy should be closely monitored with CT scans every six months. Patients with unicentric disease should receive radiological follow-up every six to 12 months, although a generally accepted regimen is not established [2,4]. Appropriate follow-up care should consider type, progression rate, clinical course and curability of the disease, and should always therefore be planned on a case-by-case basis.

**Table 2:** Overview of therapy, outcome and follow-up periods of localized retroperitoneal Castleman’s disease in the literature

**Histological variants**	**Therapy**						**Outcome**			**Follow-up**			
	**CR**	**LR**	**PR**	**RT**	**CN**	**NK**	**UNEV**	**COMP**	**NK**	**NREC**	**REC**	**Deceased**	**NK**
HV (n = 137)	77	4	4	1	0	51	67	2	67	37	0	3	97
PC (n = 9)	7	0	0	0	1	1	4	1	3	5	0	1	3
Mixed (n = 5)	5	0	0	0	0	0	3	0	2	3	0	0	2
NK (n = 37)	1	0	0	0	0	26	0	0	37	0	0	0	37

COMP, complicated postoperative course; CN, conservative; CR, conventional resection; HV, hyaline vascular; LR, laparoscopic resection; NK, not known; NREC, no recurrence; PC, plasma cell; PR, partial resection; REC, recurrence; RT, radiation therapy; UNEV, uneventful postoperative course.

## Conclusion

Castleman’s disease is a rare disorder that remains a diagnostic challenge. Radical surgical resection is considered to be the gold standard for treating the unicentric variant. This type is not associated with increased mortality as long as resection is radical and complete.

## Consent

Written informed consent was obtained from the patient for publication of this case report and any accompanying images. A copy of the written consent is available for review by the Editor-in-Chief of this journal.

## Abbreviations

CT: computed tomography; HHV-8: human herpes virus 8; HIV: human immunodeficiency virus; MRI: magnetic resonance imaging; POEMS: polyneuropathy organomegaly, endocrinopathy or edema, M-protein, skin changes.

## Competing interests

The authors declare that they have no competing interests.

## Authors’ contributions

RAR prepared and drafted the manuscript and performed the literature research. SLM edited the manuscript and performed the literature research. ID edited the manuscript and performed the literature research. SH edited the manuscript. BK performed the immunohistochemical staining and examination of the specimen, and edited the manuscript. MF edited the manuscript. HK performed the ultrasound scan and edited the manuscript. HJ was the operating surgeon and was involve in the preparation, drafting and editing of the manuscript. All authors read and approved the final manuscript.
